# The Treatment of Melancholia

**Published:** 1901-09-07

**Authors:** 


### THE TREATMENT OF MELANCHOLIA.

One is always thankful for any light on the treatment of melancholia, not only because this is one of the mental conditions which, at any rate, in its milder forms, very often comes under the care of physicians who are not specialists in lunacy, but also because any clear and well-defined line of treatment carried out with success gives hope of usefulness in other conditions besides the one for which it has been more particularly introduced. Dr. Lewis C. Bruce[1] and Dr. H. de Maine Alexander, superintendent and assistant physician respectively to the Perth District Asylum, consider that the majority of recent cases of melancholia pass through a definite course prior to recovering or becoming chronic, and this course they divide into two stages.

In the acute stage there are—1. Mental symptoms: great depression, restlessness, vivid hallucination, and sleeplessness. 2. Rapid pulse, from 90 to 120 per minute, of high tension and tending to be irregular. 3. Temperature tending to be febrile (from 99° to 100° Fahr.). 4. Urine scanty; excretion of urea deficient; trace of albumin present. 5. Tongue furred and foul; no desire for food or drink; digestive power of stomach upon coagulated albumin practically *nil*; motor power of stomach weak; bowels constipated and stools very offensive. 6. Skin dry.

In the sub-acute stage there are 1. Mental symptoms less acute; the patient generally sleeps well, hallucinations, if present, do not affect conduct. 2. Pulse regular, softer, from 70 to 80 per minute; arterial pressure less high than before. 3. Temperature never above 98·4° Fahr. 4. Urine more abundant, excretion of urea considerably increased, albumin never detected. 5. Tongue clean; appetite for food and drink returning; digestive power of stomach juice at first weak but later active; tendency to constipation less marked. 6. Skin becoming moist; sometimes perspiration profuse.

The treatment recommended is as follows:—The patients upon admission are put to bed and placed upon an exclusively fluid diet of three pints of milk and three pints of weak tea per diem, and as much water besides as they care to drink. The tea must be weak, as if strong it tends to raise the blood pressure. Persons suffering from acute melancholia are as a rule thirsty, and take this fluid diet readily. No solid food is given until the patient asks for it and complains of hunger. The results are stated to have been most satisfactory. The authors sum up the matter by saying that they believe melancholia to be a disease of disordered metabolism; that the treatment should be directed towards increasing the excretion of the waste products of this metabolism through the channels of the urinary and integumentary systems; and that they endeavour to mechanically effect this purpose by the administration of an abundant fluid dietary. They consider that the forcing of solid food, or of such food as custards upon a patient suffering from acute melancholia, is just as injudicious as would be the feeding of a patient suffering from typhoid fever exclusively upon beef-steaks.

[1] Jour. of Mental Sci., October, 1900, and Lancet, Aug. 24, 1901.

